# Medical 3D printing: methods to standardize terminology and report trends

**DOI:** 10.1186/s41205-017-0012-5

**Published:** 2017-03-17

**Authors:** Leonid Chepelev, Andreas Giannopoulos, Anji Tang, Dimitrios Mitsouras, Frank J. Rybicki

**Affiliations:** 1 Department of Radiology, University of Ottawa, 501 Smyth Road, Box 232, K1H 8L6 Ottawa, ON Canada; 2 Department of Radiology, Applied Imaging Science Lab, Brigham and Women’s Hospital, Boston, MA USA

**Keywords:** 3D printing, Rapid prototyping, Additive manufacturing, Freeform fabrication, Data integration, Terminology, Standards, Medicine

## Abstract

**Background:**

Medical 3D printing is expanding exponentially, with tremendous potential yet to be realized in nearly all facets of medicine. Unfortunately, multiple informal subdomain-specific isolated terminological ‘silos’ where disparate terminology is used for similar concepts are also arising as rapidly. It is imperative to formalize the foundational terminology at this early stage to facilitate future knowledge integration, collaborative research, and appropriate reimbursement. The purpose of this work is to develop objective, literature-based consensus-building methodology for the medical 3D printing domain to support expert consensus.

**Results:**

We first quantitatively survey the temporal, conceptual, and geographic diversity of all existing published applications within medical 3D printing literature and establish the existence of self-isolating research clusters. We then demonstrate an automated objective methodology to aid in establishing a terminological consensus for the field based on objective analysis of the existing literature. The resultant analysis provides a rich overview of the 3D printing literature, including publication statistics and trends globally, chronologically, technologically, and within each major medical discipline. The proposed methodology is used to objectively establish the dominance of the term “3D printing” to represent a collection of technologies that produce physical models in the medical setting. We demonstrate that specific domains do not use this term in line with objective consensus and call for its universal adoption.

**Conclusion:**

Our methodology can be applied to the entirety of medical 3D printing literature to obtain a complete, validated, and objective set of recommended and synonymous definitions to aid expert bodies in building ontological consensus.

**Electronic supplementary material:**

The online version of this article (doi:10.1186/s41205-017-0012-5) contains supplementary material, which is available to authorized users.

## Background

Three-dimensional (3D) printing offers plentiful opportunities for personalized and precision based interventions. The collective technologies have reduced costs and improved outcomes in essentially every industry in which they have been applied. In medicine, 3D printing has already revolutionized how we consider and treat patients in multiple clinical scenarios while offering hope for regenerative medicine [1–4].

**Fig. 1:**
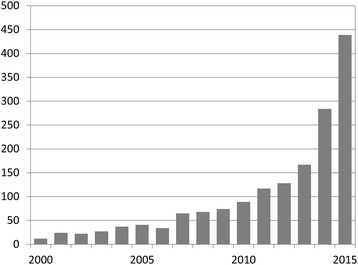
Medical 3D printing publications available in PubMed by publication year, from 2000 to 2015

While research relating on 3D printed models of patient anatomy and pathology has undergone exponential growth (Fig. 1), there is little data regarding publication metrics for 3D printing stratified by organ section, imaging modality used to produce the models, or the 3D printing hardware used. Furthermore, while 3D printing has been used globally, there is little organized data regarding the geographic distribution of 3D printing publications. An understanding of the major directions and stakeholders in this domain is an indispensable step to help build a collaborative dialogue.

An integrated assessment of available literature is crucial to establish a common, medicine-specific, vocabulary needed to facilitate collaborative research, knowledge integration, and ultimately reimbursement. Little formal consensus exists in the literature on fundamental concepts, including the naming of the field of medical 3D printing, with domain-specific publications using alternative terms such as “rapid prototyping” and “additive manufacturing” among many others. In light of the rapid growth in the number and diversity of research efforts in medical 3D printing, it is imperative to standardize specific terminology in the peer-reviewed literature before knowledge “silos” create significant barriers to collaborative efforts.

Centralized medical publication repositories and data mining technologies have enabled rapid large-scale analyses of entire scientific domains. Natural language processing and semantic web technologies allow medical publications to be recast as machine usable knowledge, facilitating objective integration between medicine and other disciplines such as chemistry, biology, and epidemiology [5–7]. These technologies provide a tremendous opportunity in enabling rapid and objective integration of all published medical 3D printing data.

The purpose of this study is to develop and apply an objective scientific basis in order to standardize 3D printing terminology that will facilitate scientific, clinical, and regulatory communications. Using this basis, we present the evaluation of 3D printing research trends within published work to date, organized by medical discipline as well as geographic distribution. We then derive the dominant terminology within the domain to propose a common term to represent a collection of technologies that produce physical medical models. With this approach, we intend to facilitate more detailed analyses to further define trends and focus studies, better integrating 3D printing technologies and cultivating collaboration, better recognition, and ultimately supporting reimbursement.

## Methods

Published papers pertaining to medical 3D printing up to January 2016 were identified within PubMed using the following search query:

(rapid[All Fields] AND prototyping[All Fields]) OR (additive[All Fields] AND manufacturing[All Fields]) OR (“printing, three-dimensional” [MeSH Terms] OR (“printing” [All Fields] AND “three-dimensional” [All Fields]) OR “three-dimensional printing” [All Fields] OR (“3d” [All Fields] AND “printing”[All Fields]) OR “3d printing”[All Fields]) AND “humans” [MeSH Terms]

All full text publications were manually retrieved and screened to ensure that papers having only marginal relationship to medical 3D printing were not included. Specifically, screening included a rapid survey to ensure either of the major terms and derivatives were present with simple text search-based screening. Papers yielding no matches for any of the direct or synonymous terms to those searched above in the full text were discarded. The remaining papers were analyzed using software developed within our group. The purpose of this software was threefold: i) to create a text corpus (i.e., collection) amenable to computer query and analysis, ii) to discover recurrent domain-specific terms, and iii) to analyze publication metadata such as date and geographic location in relation to domain-specific term use. To accomplish these tasks, the software first converted full text papers from PDF to plain text, extracted available article metadata, and isolated article text by removing references. The software subsequently extracted sentences, phrases, and individual terms to generate a text corpus. This text corpus then underwent the following four separate analyses.

### Analysis 1: Standardizing terminology for 3D printing

Based on our observations and preliminary analyses, we identified three major terms, plus variants, that are used to describe the generation of 3D medical models: “3D Printing”, “Rapid Prototyping”, and “Additive Manufacturing”. Within a given peer-review publication, variations were used to determine the dominant term; dominance was established by comparison of simple tallies of the three terms within the full text of a given paper to identify the most frequently used term. Then, the year of publication was extracted for each paper and the number of publications was tallied by year that they appeared in the literature, beginning with 2002.

### Analysis 2: Generalized overview of 3D printing literature concept clusters

**Fig. 2:**
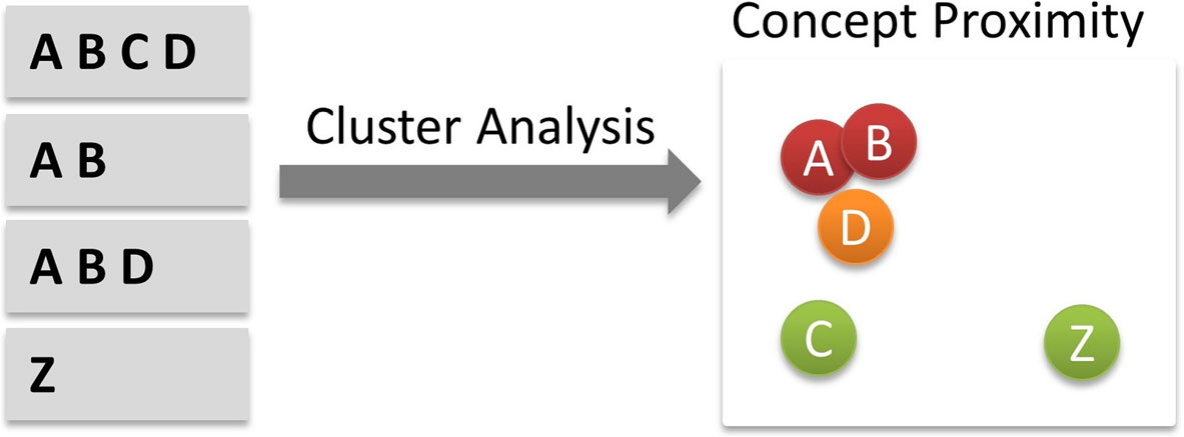
Schematic representation of concept cluster analysis. Here, a literature corpus is represented on the left, with the concepts “A” and “B” always encountered together in published literature. This is reflected in these concepts closely clustering together on a proximity map. Concept “Z”, on the other hand, is isolated, and is well outside of the “AB” cluster on the map. Colors denote total concept use, with red reflecting maximal and green minimum use

Medical Subject Headings (MeSH), used by PubMed to categorize publications by major medical topics, were used for the identification of concept clusters within the available literature. A concept cluster refers to a collection of related terms, with concept proximity defined as the strength of term relationship reflected in the number of papers for which terms in the cluster co-occur (Fig. 2). A concept cluster map for all 3D printing literature was generated by applying the PubPlus [8] software to the entire cohort of papers and their associated MeSH annotations obtained directly from PubMed as a result of the search and selection above. Resultant cluster maps were visualized using VOSviewer [9] software.

### Analysis 3: Stratification of 3D printing publications by anatomical site, imaging modality, and 3D printing technology

Because a thorough manual examination of available MeSH terms yielded neither a sufficient level of granularity nor sufficient breadth of annotations necessary for this analysis, custom text mining was implemented. Individual recurrent words and phrases extracted by our software were collected. Term usage was tallied as described above, and terms exceeding an arbitrary threshold of 10 uses within the corpus were selected. Approximately 2000 common words in the English language identified after applying this threshold but without any direct significance in medicine and anatomy were excluded. The remaining terms were manually screened to identify 500 terms describing anatomy, hardware/software types and vendors, applications, and printing materials. These selected terms were then manually grouped into appropriate classes for simplicity of presentation. For example, “mitral valve” and “tricuspid valve” and their automatically identified plural forms were grouped into the “cardiac” discipline. Major imaging modalities, fabrication technologies, and application domains were also included. Papers were then annotated with a dominant class assignment (e.g. “cardiac” rather than “orthopedic”) by simple term majority. Finally, the text corpus was iteratively screened for dominant class colocation, providing counts of papers in each relevant binary class combination (e.g. “cardiac” discipline with “education” application). Sample representative terms for each discipline and category are included in Additional file 1: Appendix 1.

### Analysis 4: Stratification of 3D printing publications by country of origin and date

Metadata of all articles included in the corpus was obtained by parsing the XML of the screened result of the PubMed query directly, including author affiliation (where available) and publication date. Using this information, the country of origin of the first author was obtained directly from XML. When country of origin was not explicitly specified, it was identified through geographical matching of the specified portion of author addresses to countries. These data were tabulated and displayed on a map and in chronological order for all represented countries using R version 3.3.0 [10].

### Analysis 5: Evidence of terminological isolation for top-level terms

To establish evidence of terminological isolation for the terms referring to the medical 3D printing research domain, a modification of Analysis 3 was used. Using the established research disciplines of Analysis 3, the absolute number of occurrences of the three major top-level terms (‘3D printing’, ‘rapid prototyping’, and ‘additive manufacturing’) and their variants were tallied for each discipline. The absolute counts of these terms were converted to relative use percentages by setting the ‘3D printing’ term as the reference standard at 100%. While concept clusters from Analysis 2 were available for demonstration of term usage disparity, usage stratification based on the broad medical fields discussed in Analysis 3 was felt to represent a more appropriate reflection of the broad diversity of medical research in each discipline.

## Results

### Analysis 1: Standardizing terminology for 3D printing

**Fig. 3:**
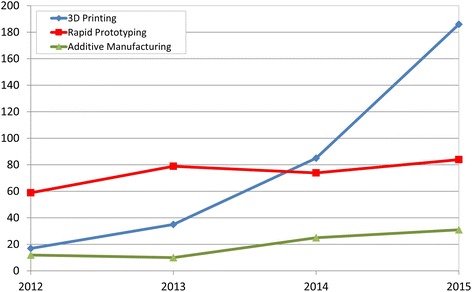
Publications primarily referencing “3D printing”, “rapid prototyping”, or “additive manufacturing” terms in the period from 2012 to 2015

Beginning in 2014, the term “3D printing” surpassed “rapid prototyping” and then proceeded to realize widespread growth in 2015. By 2015, “3D printing” accounted for 64% of all papers in the domain, with “rapid prototyping” and “additive manufacturing” accounting for 29 and 11%, respectively (Fig. 3). Of note, the percentages do not sum to 100% because the methodology allowed co-dominance if an individual paper had equal counts of two or more terms.

### Analysis 2: Generalized overview of 3D printing literature concept clusters

**Fig. 4:**
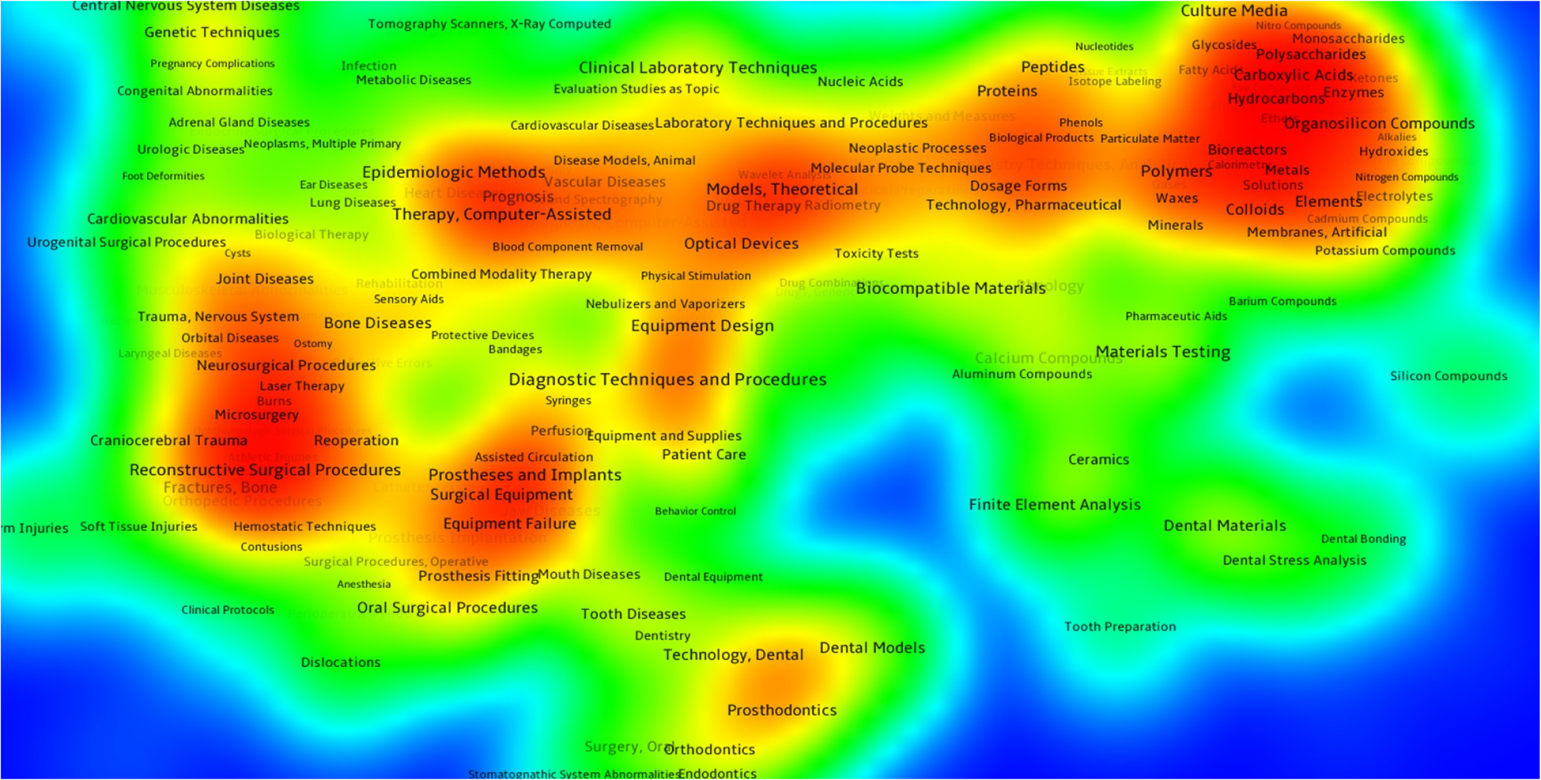
Heat map for concept clusters within 3D printing literature. Red corresponds to a large number of highly clustered and related concepts, while blue represents absence of relationships

**Fig. 5:**
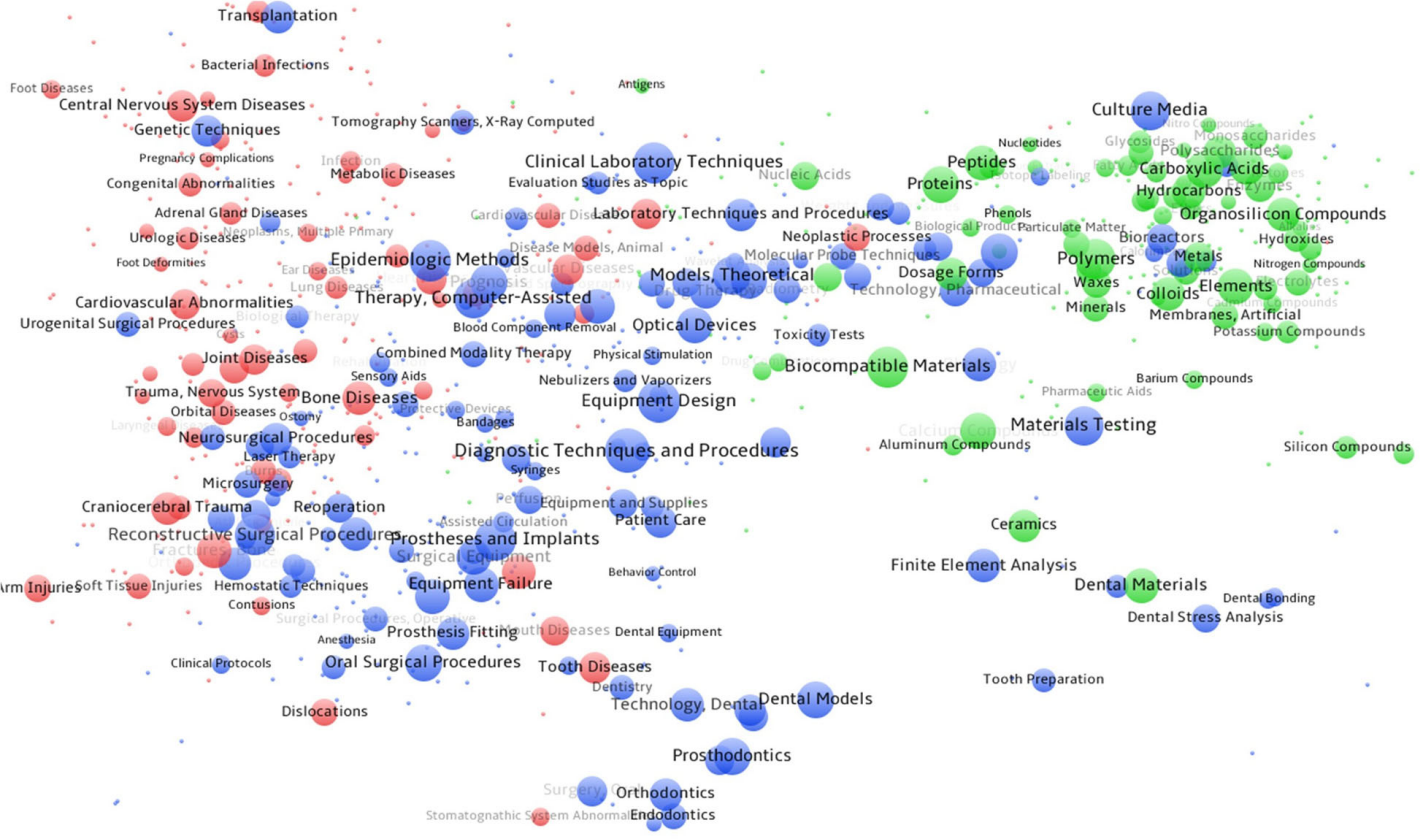
Concept clusters within 3D printing literature arranged as individual MeSH categories and clustered by distance within the literature. Colors correspond to major MeSH categories: red represents “Diseases”, green corresponds to “Chemicals and Drugs” and blue represents “Analytical, Diagnostic and Therapeutic Techniques and Equipment”. Circle size represents the relative number of papers annotated with a particular MeSH term, while distance reflects concept proximity

We illustrate MeSH term clusters in two ways (Figs. 4 and 5). First, we provide a rapid visual overview of concept clusters (red), denoting closely linked concepts in published literature (Fig. 4). For example, the upper right hand cluster in Fig. 4 refers to bioprinting and materials research. The same cluster in Fig. 5 appears in green, highlighting its major MeSH category (“Chemicals and Drugs”). These display formats enable rapid visual analysis of major research directions and discipline clusters. Some of the more prominent research ‘islands’ include surgical equipment, dental technologies, disease modeling, and material development fields.

### Analysis 3: Stratification of 3D printing publications by anatomical site, imaging modality, and 3D printing technology

**Fig. 6:**
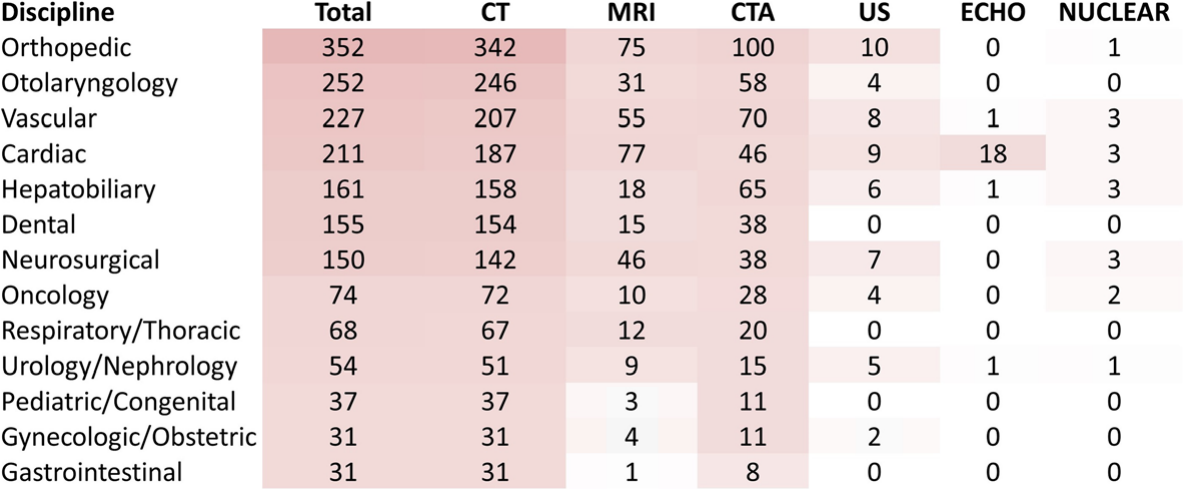
Publications in 3D printing per medical discipline and the supporting imaging modalities used in each discipline. Please note that discipline and modality co-dominance is possible within a publication, leading to row sums exceeding the total provided

**Fig. 7:**
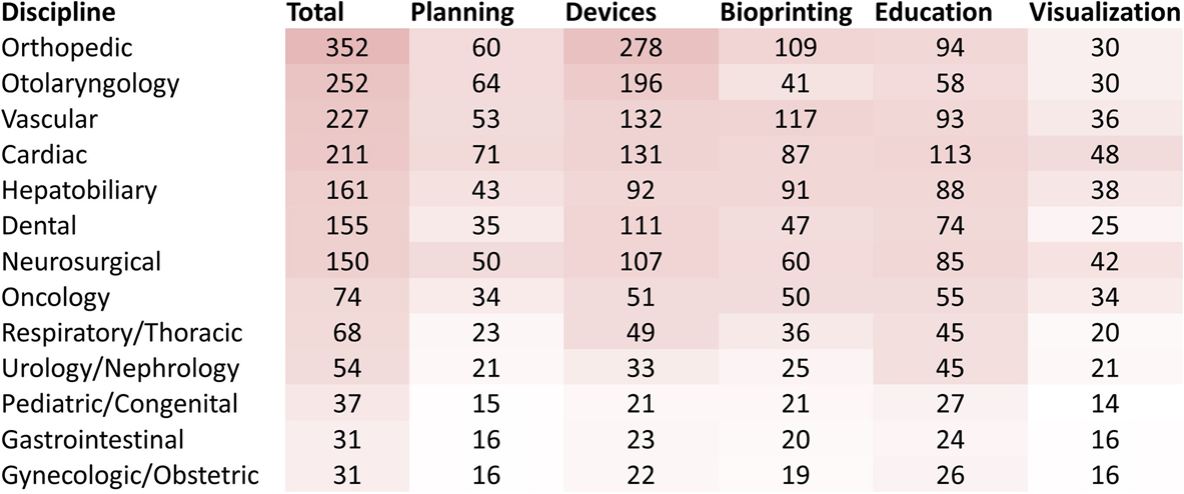
General application categories in specific medical disciplines

**Fig. 8:**
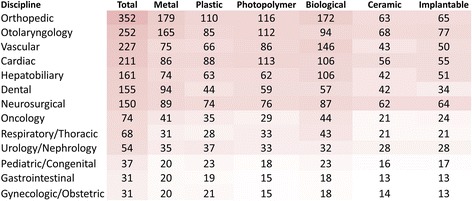
Printing technologies employed in major disciplines and applications, stratified by main manufacturing process and material category

CT based 3D printed models greatly outnumber other cross-sectional imaging modalities capable of volumetric reconstruction (Fig. 6) except in cardiovascular medicine where MRI and echocardiography are comparable. Orthopedic applications are among those most published (Fig. 7), including surgical planning and the creation of patient-specific orthopedic implants. Other common disciplines include otolaryngology, vascular and cardiac medicine, likely reflecting a high use of 3D printed models to plan and teach higher risk interventions for complex clinical scenarios. The leading applications in 3D printing of organs and tissues (bioprinting) are within vascular, skeletal, hepatic, and cardiac domains, in the order of significance (Fig. 8).

### Analysis 4: Stratification of 3D printing publications by country of origin and date

**Fig. 9:**
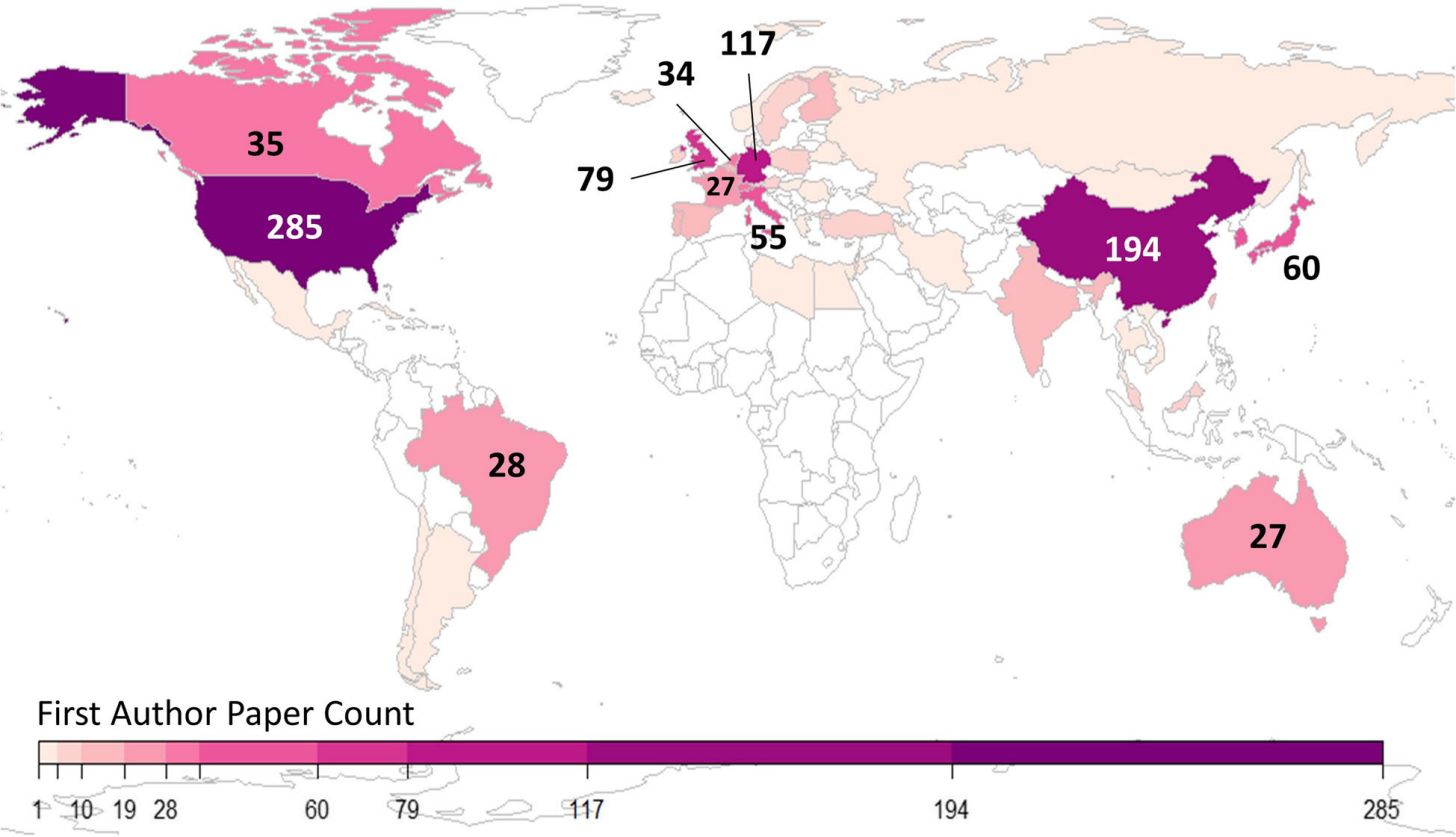
Global medical 3D printing activity, by the country of origin of the first author. Nations with over 25 publications are annotated on the map, with the exception of Switzerland, with 32 first author publications

Medical 3D printing enjoys widespread attention within the global scientific community (Fig. 9). While Europe has the most publications as a continent (374), national major leaders include the United States with 285 first author publications, followed by China (194) and Germany (117). Of a total of 1195 papers available for this study, 151 did not have adequate nation of origin assignments available within the metadata.

### Analysis 5: Evidence of terminological isolation for top-level terms

**Fig. 10:**
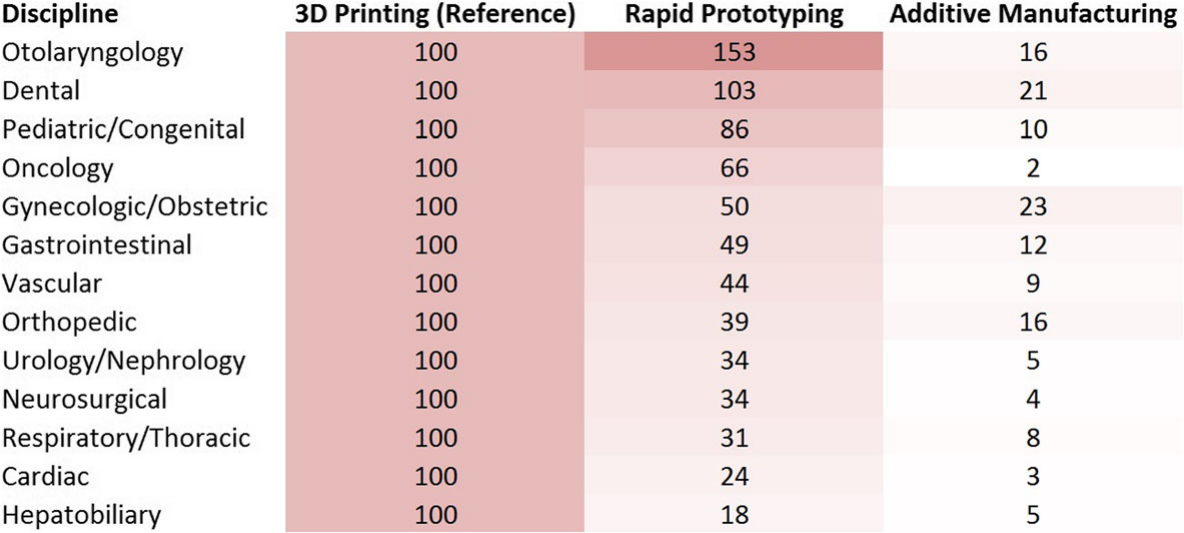
Usage of ‘additive manufacturing’ and ‘rapid prototyping’ in relation to ‘3D printing’, in percent. Although for the vast majority of fields, ‘3D printing’ is the relatively more frequently used term, dentistry and otolaryngology provide evidence for nascent terminological isolation, with ‘rapid prototyping’ being the more frequently used term

While the vast majority of medical fields prefer the term ‘3D printing’ as the major term to refer to the research domain applications in otolaryngology and dentistry demonstrated 53 and 3% greater predisposition to use the term ‘rapid prototyping’ respectively (Fig. 10).

## Discussion

Our formalized methodology supports the standardization of terms derived from literature consensus. We demonstrate the development of closely linked publication concept clusters (Figs. 4, 5) that reflect major research directions but can also become fertile grounds for terminological isolation in the absence of a commonly accepted language. Furthermore, we provide evidence of the disparity in terminological use at the level of entire medical disciplines (dentistry, otolaryngology) even for the most important top-level terminology (Fig. 10). These differences have likely arisen from historical chance factors, since qualitative assessment of imaging modalities (Fig. 6), applications (Fig. 7), and specific printing technologies (Fig. 8) does not reveal a significant disparity to justify the observed terminological use difference. To avoid fragmentation of research efforts and terminological isolation in the setting of a lack of objective reasons for terminology use difference, common literature-supported terminology should be established.

Our analysis establishes the dominance of the term “3D printing” to represent a collection of technologies that produce physical medical models. The success of medical 3D printing technologies will be more readily pursued by universally adopting the term “3D printing” that already encompasses most of the total use in the recent literature. We thus propose that “3D Printing” be formally adopted.

Both “rapid prototyping” and “additive manufacturing” have historical significance. The term rapid prototyping originally denoted all technologies that could rapidly produce a physical prototype of a virtually designed object. *Additive manufacturing* was established to distinguish it from the dominant Computer Numeric Control (CNC) subtractive methods, where raw materials in sheet or block form are secondarily processed with numerically controlled cutting and milling tools to *subtract* the unnecessary parts, leaving behind a whole prototyped object, or parts of the object that could be assembled into one. *Rapid prototyping* therefore encompassed *additive manufacturing* and *subtractive manufacturing* [11]. Additive manufacturing then encompassed technologies ranging from electron beam melting to fused filament fabrication, with “3D printing” abbreviated as 3DP originally relegated only to a set of technologies emerging at the Massachusetts Institute of Technology that produced models by binding powder using an adhesive, much like an inkjet printer [12].

Our analysis demonstrates that over time, the meaning of these technologies has evolved in medicine to first make 3D printing nearly synonymous and interchangeable with the other two terms. Over time, “3D Printing” surpassed all others in literature. We recognize that more data can influence the use of terminology over time. We also recognize that, in theory, there may be specific medical uses for which the term “3D Printing” may not be scientifically accurate. In such rare cases, the most accurate terms should be used. However, the adoption of the term 3D Printing should be used for other cases so as to provide standardization and the ability of the field to mature with a standard lexicon.

Our analysis did reveal alternative terms, including, “*solid freeform fabrication*”. While this term has significance in general industrial applications, it is not significantly used relative to the other three terms in medical applications. Within our study, this term was observed in minority niche use, primarily within bioprinting research, specifically in relation to the 3D printing of hydrogels and tissue scaffolds (Additional file 2: Appendix 2). As demonstrated in concept cluster analysis (Figs. 4 and 5), bioprinting is a vibrant field that is relatively distanced from the numerous other concept clusters in the 3D printing domain. The use of the relatively rare term “*freeform fabrication*” to describe 3D printing technologies may therefore be regarded as another line of evidence of terminological fragmentation and knowledge “siloization” in development.

One limitation of this work is that many 3D printed models used in medicine do not reach the peer-reviewed literature, and “niche” laboratories are contributing largely to patient care, but to date are not recognized by the scientific community. Thus, the actual practice may not be faithfully reproduced, even with an exhaustive analysis of the literature. Within these limitations, our data demonstrates that the greatest impact made by 3D printing so far is in musculoskeletal and cardiovascular domains where it is used in support of procedure planning and device creation in support of operative interventions and postoperative recovery. Bioprinting is also evolving rapidly, not only for printing skeletal structures for which allografts were used for decades prior to the advent of widespread 3D printing, but also for expanding the field to include printing soft tissues such as the liver and the supporting vasculature.

While we have established evidence for “3D printing” as the discipline name, the remainder of the domain terminology is not yet organized. This requires not only a deeper analysis using the methodology described here, but also a shift in the perception of publication value in medical 3D printing. Guidelines must be established for reporting 3D printed models, including those that call for complete description of the technologies used [13]. Individual 3D printed models and their applications should be recognized as case reports in medical literature, and models themselves should be stored in repositories using a single common format, in a manner similar to PubChem and the Protein Data Bank in chemistry and biochemistry, respectively.

The methodology described has impact beyond the identification of “3D Printing” as the dominant recommended terminology in medicine. This objective approach should be used as a foundation of a discussion to obtain a single ontology, that is, a formalized collection of standard terms for all medical 3D printing, including terms developed in the future. That is, we can organize and describe 3D printing terminology into logically consistent categories that can be referenced, followed, and extended as the technology grows. The most important terms will become part of the frontline medical lexicon for 3D printing as the field grows, and this ontology would be used by computers to standardize and facilitate data analyses and integration, thereby avoiding research duplication and confusion. The risk of not formalizing terminology in this stage of rapid growth is “siloization” and fragmentation of research and clinical applications. It will be increasingly important to develop, validate, and adhere to standards throughout 3D printing, beginning with and including those related to terminology.

## Conclusion

Medical 3D printing continues rapid expansion in scope and number of publications. Within this rapid expansion, however, evidence of terminological research isolation has emerged. We demonstrate an objective methodology for rectifying terminological discrepancies and apply it to identify the dominant name for medical 3D printing research. The term “3D Printing” is the dominant term in the domain and should be formally adopted as the principal discipline name moving forward, to the exclusion of other synonyms and alternative names where appropriate. This analysis should be expanded to other terms and controversial concepts and used to support expert consensus in establishing a common ontology and a means of objective unbiased discussion of the terminology needed to enable collaboration, seamless knowledge integration, and medical reimbursement.

## Additional files

Additional file 1: Appendix 1. Sample representative terms for major disciplines, technologies, and applications. (DOCX 16 kb)

Additional file 2: Appendix 2. Use of “Freeform Fabrication” Terminology. (DOCX 20 kb)

## Abbreviations

3D: Three dimensional
CNC: Computer numeric control
CT: Computed tomography
CTA: Computed tomography angiography
MeSH: Medical Subject Headings
MRI: Magnetic resonance imaging
US: Ultrasound
