# Supplementary material for: Medical 3D printing: methods to standardize terminology and report trends
Source: 3D Print Med. 2017 Mar 17;3:4. doi: 10.1186/s41205-017-0012-5 (PMC6036766; doi:10.1186/s41205-017-0012-5)
Supplement: Supplementary file 1 — Sample representative terms for major disciplines, technologies, and applications. (DOCX 16 kb) [file 41205_2017_12_MOESM1_ESM.docx]

Appendix 1

Sample representative terms for major disciplines, technologies, and applications

Although not all anatomical and thematic features pertinent to each discipline are found within these lists, the terminology presented here is derived from the highest-scoring recurrent terms in the literature. Therefore, e.g. the hepatobiliary section contains mention of focal nodular hyperplasia without very many additional pathologies of the hepatobiliary system. Thus, underrepresented fields are bound to have relatively fewer descriptors than well-represented ones. Furthermore, terms used were selected to be specific to a discipline. Thus, ‘disk’ is not used for orthopedics. ‘Intervertebral disk’ may have been used instead, but the occurrence of the term ‘intervertebral’ is so low that this term does not make the cutoff of a minimum of 100 mentions.

**1. Disciplines**

Cardiac: cardiac, heart, mitral, tricuspid, pulmonary valve, aortic valve, coronary artery, coronary, left ventricle, right ventricle, atrium, papillary, ventricular septal, atrial septal, cardiac, heart, aortic valve, coronary artery, valve replacement, pulmonary artery, ascending aorta, ventricular septal, mitral valve, cardiac tissue, pulmonary valve, tricuspid, ventricular outflow, septal defect, valve implantation, cardiac anatomy, cardiac structures, aortic annulus, valve stenosis, vena cava, aortic stenosis, artery bypass, myocardial infarction, cardiac model, pulmonary atresia, aortic regurgitation, cardiovascular anatomy, prosthetic mitral, cardiopulmonary bypass, interactive cardiovascular, papillary muscles, pulmonary trunk, pulmonary venous, cardiovascular diseases, interventional cardiology, myocardial tissue, prosthetic valve, cardiac tumors, valve leaflets, coronary angiography, valve repair, valve scaffolds, bioprosthetic valves, cardiac regeneration, cardiovascular tissue, valve diseases, mitral annuloplasty, myocardial perfusion.

Vascular: vascular, aorta, vena cava, svc, ivc, artery, vein, arteriovenous, avm, aneurysm, hemangioma, spleen, artery stenosis, atherosclerosis, embolism, embolus, thrombus, spleen, vascular, aortic arch, vascular structures, vascular anatomy, transcatheter aortic, vascular networks, abdominal aorta, aortic aneurysm, descending aorta, superior vena, inferior vena, aortic coarctation, subclavian artery, thoracic aorta, portal vein, aneurysm models, aortic dissection, endovascular stent, mesenteric artery, renal artery, celiac artery, celiac trunk,carotid.

Gastrointestinal: mesentery, rectum, colorectal, jejunum, gastric, gastrointestinal, colon, rectum, sigmoid, colonic diverticulum, esophagus.

Hepatobiliary: hepatobiliary, hepatic, biliary, liver, gallbladder, cholangiocarcinoma, focal nodular hyperplasia, pancreas, pancreatitis, liver, liver transplantation, hepatic artery, hepatic vein, bioartificial liver, hepatic segment.

Orthopedic: orthopedic, orthopaedic, bone, osteotomy, joint, fracture, musculoskeletal, cartilage tissue, articular cartilage, fibula, iliac crest, femoral condyle, le fort, pedicle screw, hip arthroplasty, distraction osteogenesis, distal radius, orthopedic implants, thoracic spine, acetabular defects, alt flap, artificial semi-knee, cervical vertebra, condylar osteochondroma, lumbar spine, proximal femur, osteotomy, vertebra, tendon, glenoid,pelvis.

Respiratory/Thoracic: respiratory, thoracic, airway, thorax, mediastinum, lung, trachea, airway splint, tracheal splint, tracheal reconstruction, tracheobronchial splint, mainstem bronchus, tracheal graft, tracheal compliance, tracheobronchial chondromalacia.

Neurological: neurosurgery, brain, neurological, central nervous, skull, brain, carotid artery, spinal cord, cranial defect, cervical spine, skull model, cerebral aneurysms, cortical neuron, skull defect, defect reconstruction, optic nerve, anterior communicating, intracranial aneurysms, cranial, craniectomy, neuron.

Gynecologic and Obstetric: gynecologic, obstetric, fetus, gestation, trimester, ovary, fallopian, endometrium, uterus, vagina.

Oncology: oncology, cancer, neoplasm, tumor, tumour, sarcoma, adenocarcinoma, carcinoma, myeloma, leukemia, lymphoma, ewing sarcoma.

Genitourinary/Renal: urology, urologic, nephrology, renal, kidney, calyx, ureter, bladder, urethra, prostate, renal calculi, prostate, bladder outlet.

Otolaryngology: otolaryngology, maxilla, nose, pharynx, maxillofacial, mandible, mandibular reconstruction, temporomandibular joint, facial contour, mandibular angle, facial prosthesis, facial asymmetry, mandible reconstruction, orthognathic surgical, mandibular defects, mandibular fractures, lower jaw, nasal prosthesis, auricular prosthesis, auricular reconstruction, bony reconstruction, facial symmetry, inferior turbinate, mandible defect, auricular cartilage, cochlear implant, dentofacial deformities, lumbar pedicle, orbital reconstruction, mandibular distraction, mandibular incisors, narrowing genioplasty, facial.

Dentistry: dental, teeth, tooth, incisor, oral, orthodontic, dental appliance.

Pediatric/Congenital: pediatric, paediatric, congenital, developmental, neonate, infant, pediatric cardiac, congenital heart, pediatric cardiology.

**2. Applications**

Planning: surgical planning, preoperative planning, virtual planning.

Personalized Instruments: surgical template, drilling jig, cutting template, personalized fixation plate, graft, implant, prosthesis, screw.

Bioprinting: bioprinting, biological printing, tissue engineering, bioink, organ printing.

Education: education, learner, student, teaching, trainee, resident.

Anatomy Visualization: complex anatomy, anatomy visualization, anatomical relationship.

**3. Materials**

Plastic: termoplastic, acrylonitrite butadiene, polycaprolactone, makerbot, polylactic acid, high-impact polystyrene, polyurethane, nylon, peek, paek, thermoplastic.

Photopolymers: stereolithography, polyjet, photopolymer, photopolymerization, liquid resin.

Metals: metallic, alloy, titanium, steel, bronze, gold, nickel, aluminum, aluminium, metal.

Ceramics: ceramic, clay, porcelain.

Bioprinting Materials: bioprinting, stem cell, hydrogel, fibroblast, bio-ink, cell-laden alginate, polycaprolactone, hydroxyapatite, pluripotent, mesenchymal, osteoblast, organ printing.

Biocompatibles: biocompatible, implantable.
