# Supplementary material for: Medical 3D printing: methods to standardize terminology and report trends
Source: 3D Print Med. 2017 Mar 17;3:4. doi: 10.1186/s41205-017-0012-5 (PMC6036766; doi:10.1186/s41205-017-0012-5)
Supplement: Supplementary file 2 — Use of “Freeform Fabrication” Terminology. (DOCX 20 kb) [file 41205_2017_12_MOESM2_ESM.docx]

Appendix 2.

Use of “Freeform Fabrication” Terminology

The following passages are direct excerpts from the text corpus used in this study. Detailed assessment of each statement reveals a strong bioprinting bias for the use of the term “freeform fabrication”, as well as tones of the evolution of the meaning of this term from its origins of a more general way of describing 3D printing and related technologies. In the vast majority of cases, however, “3D printing” is implied in lieu of “freeform fabrication”.

- in order to fabricate 3d scaffolds containing bio-organic polymers, e.g., collagen as supporting fiber, and avoiding any denaturating temperatures, the solid freeform fabrication (sff) technique [128] coupled to the indirect 3d printing technique [129] was developed
- this range of techniques, also referred to as solid freeform fabrication or additive biomanufacturing, enables precise positioning of cells and biomaterials in 3d with finely tuned internal and external architectures, while being customizable to patient-specific needs
- hydroxyapatite and bis-gma based acrylate were prepared by a new technique namely 3dp which is one of freeform fabrication technologies
- a new technique, so-called on-demand 3d freeform fabrication (ff), has also emerged to construct tissues and organs in 3d, on-demand
- a successful 3d scaffold made of plla and plga was prepared using this laminating technique, and the pores were interconnected forming a continuous pore structure.53 rapid prototyping to overcome the limitations of conventional fabrication methods, rapid prototyping (rp) technologies (a.k.a solid freeform fabrication or additive manufacturing technologies) have been developed and increasingly applied in bte
- agarose is a suitable material for the 3d freeform fabrication of large tissues by submerged printing
- although a number of studies have shown that wet-spun non-woven scaffolds possess a 3d structure with high and interconnected porosity suitable for te purposes and that they can be easily functionalized by loading with bioactive agents, the methods employed for the preparation of wet-spun assemblies do not allow an accurate control over scaffold external shape and internal morphology. additive manufacturing techniques additive manufacturing (am) techniques (also known as solid freeform fabrication techniques) involve the layer-by-layer building of porous structures from 3d model data by successively fabricating microstructured layers.2 as shown in figure 1, a 3d computer solid model, obtained using medical imaging techniques or directly designed in cad software, is expressed as a series of cross-sectional layers with predeﬁned thickness. the data are then implemented to the am machine, which reproduces physically the model through an additive process
- although angiogenic factors can induce sprouting of vessels into ischemic tissue, this slow (0.1mmper day) ‘‘extrinsic vascularization’’ can take months to perfuse large organs, eliciting necrosis developed through techniques including soft lithography, cast hydrogels, coculture, and solid freeform fabrication (sff). recent advances in these methods have made strides toward building 3d cons-approach to creating sacrificial templates with vascular-like branching
- biopolymer deposition for freeform fabrication of hydrogel tissue constructs
- bioprinting technology, which applies freeform fabrication methods to deposit scaffold materials and cells to form digitally deﬁned 3d structures, constitutes a basically novel approach to approach this so far unsolved issue: there are various types of 3d manufacturing techniques addressing a broad range of structure sizes and several have already been used for biofabrication purposes as well
- cate can be defined as the application of enabling computer-aided technologies, including computer-aided design (cad), image processing, computer-aided manufacturing (cam) and rapid prototyping (rp) and/or solid freeform fabrication (sff) for the modelling, designing, simulation and manufacturing of biological tissue and organ substitutes
- cate integrates advanced imaging technologies such as computed tomography (ct) or magnetic resonance imaging (mri), computer-aided design (cad) technology, and rapid prototyping (rp) and/or solid freeform fabrication (sff) technology with tissue engineering applications
- collagen scaffolds were fabricated using a solid freeform fabrication (sff) system (solidscape t66, solidscape, us), the details of which have been described elsewhere. in brief, the manufacturing process (figure 1) involves (1) computer aided design and fabrication of a sacriﬁcial negative mold; (2) casting of the collagenous solution into the negative mold and freezing at 2308c; (3) dehydration and dissolving away the negative mold to create a predeﬁned microchannel network; (4) critical point drying and crosslinking of the scaffolds
- conventional freeform fabrication has already been adapted for printing a variety of sophisticated 3d tissue engineered scaffolds from synthetic biodegradable polymers with sequential bioreactor-based cellularization (two step biofabrication process) making them especially suitable for fabrication of hard tissues
- delivery of bioink particles with this technology was rapid, accurate and assured maximal cell density, while showing minimal cell damage that is often associated with other solid freeform fabrication-based deposition methods focused mostly on printing cells in combination with hydrogels
- developed an solid freeform fabrication-based 3d scaffold consisting of poly (lactic-co-glycolic acid) (plga) grafted with hyaluronic acid, in which an intact bmp-2/poly (ethylence glycol) complex was encapsulated. in addition, lee et al
- direct freeform fabrication of seeded hydrogels in arbitrary geometries
- due to the rheological as well as the viscoelastic properties, a wide range of scaffolds can be fabricated, not only via electrospinning but also by gravity spinning, as well as by solid freeform fabrication
- effects of dispensing pressure and nozzle diameter on cell survival from solid freeform fabrication-based direct cell writing
- further research is necessary to optimize the material system and the engineering design of the phantom fdm technique for freeform fabrication of 3-d tissue-simulating phantoms with high productivity and fidelity
- in order to reduce costs and provide faster and better service to dental patients, a new dental restoration method multi-material laser densification (mmld), based on the principle of solid freeform fabrication (sff), is being developed
- microﬂuidic device for isolation and detection of ha–qds was fabricated using three-dimensional (3d) printing, which is an example of additive manufacturing or solid freeform fabrication technology
- multi-layered biomimetic skin can be constructed through3d freeform fabrication skin tissue regeneration fibroblasts keratinocytes collagen hydrogel
- on-demand three-dimensional freeform fabrication of multi-layered hydrogel scaffold with fluidic channels
- on-demand three-dimensional freeform fabrication of multi-layered hydrogel scaffold with fluidic channels
- our experiment results demonstrate the technical feasibility of using the fdm technique for freeform fabrication of phantoms that simulate tissue structural and optical heterogeneities
- rapid prototyping (rp), also termed “solid freeform fabrication (sff),” is a recent technology based on the advanced development of computer and manufacturing
- recently, solid freeform fabrication (sff) has played important roles in the fabrication of negative mold with desirable architecture
- review: the application of solid freeform fabrication technology to the production of tissue engineering scaffolds
- scaffolds with very complex 3-d architectures and predetermined size, shape and porosity can be fabricated using solid freeform fabrication (sff) through computer-aided design (cad)
- scanning electron micrographs of 300 mm diameter multicellular spheroids ofbioink particles with this technology was rapid, accurate and assured maximal cell density, while showing minimal cell damage that is often associated with other solid freeform fabrication-based deposition methods focused mostly on printing cells in combination with hydrogels
- solid freeform fabrication (sff) and 3d printing are two of the more popular rapid prototyping techniques that are capable of generating multi-material and multi-cellular anatomical constructs
- solid freeform fabrication (sff), also known as rp, or layered manufacturing (lm) can offer a possibility to fabricate scaffold with customized external shape and reproducible internal morphology
- solid freeform fabrication of three-dimensional scaffolds for engineering replacement tissues and organs
- solid freeform fabrication sff technologies create laminated 3d structures from numerical models
- the advance of solid freeform fabrication has signiﬁcantly improved the ability to prepare cultures with precise geometries using computer aided designs and data from (medical) imaging
- the bio-degradable and biocompatible solid freeform fabrication (sff) scaffolds have been used in tissue fabrication in vitro
- the development of freeform fabrication technology has become a promising tool for the manufacturing of biological scaffolds for tissue regeneration and stem cell engineering
- the integration of scaffold computational design and freeform fabrication techniques presented here could prove highly useful for the construction of scaffolds that have anatomy speciﬁc exterior architecture derived from patient ct or mri data and an interior porous architecture derived from computational design optimization
- therefore, further improvement of the fdm process and optimization of the material system are required for freeform fabrication of a 3-d brain model with high structural fidelity in the future
- these include, among others, solvent casting combined with particulate leaching, freeze drying, gas foaming, melt moulding, ﬁbre bonding, phase separation techniques, electrospinning and additive manufacturing (am) techniques (also known as solid freeform fabrication techniques)
- they demonstrated freeform fabrication of tubular systems with capillary dimensions using, for example, the above-mentioned pthfbased materials
- this emerging field encompasses computer-aided design (cad), image processing,manufacturing and solid freeform fabrication (sff) for modelling, designing, simulation and manufacturing of biological tissue and organ substitutes
- this is one type of freeform fabrication technology that offer the ability to readily produce complex-shaped and personalized implants suitable for individual patients
- three-dimensional (3d) freeform fabrication (ff)multi-layered culture of human skin
- to facilitate standardized calibration and performance evaluation of medical optical devices, we develop a three-dimensional fuse deposition modeling (fdm) technique for freeform fabrication of tissue-simulating phantoms
- to properties of 3d printed hydrogels direct the differentiation of msc 753 make use of a 3d bioprinter for the creation of tissue-like objects it is essential to achieve a successful cell-supporting material printability, allowing for the freeform fabrication of individual structures
- used this approach to print hepg2 liver cells within methacrylated gelatin rods.5 stereolithography and projection patterning stereolithography is a long-used solid freeform fabrication technique that employs a reservoir containing photocurable polymer solution or resin, a laser with x–y control, and a stage or fabrication platform with vertical control
- yet, the stacking of droplets or strands is difﬁcult to control and without a stabilizing bath as in the cacl2-alginate system, adequate supporting materials and structures are needed for freeform fabrication processes
- development of a 3d bioprinter to afford the flexibility of controlling the geometrical microand macro-cellular environments and the cell-to-cell interactions in 3d: we have developed a robotic bioprinting platform based on the 3d solid freeform fabrication technology
- freeform fabrication and precise cell placement in 2d and 3d
- freeform fabrication techniques are aided by the inherent ability of cells and tissue to self-assemble, an excitingly new way to construct tissues layer by layer
